# Upconversion luminescence and optical thermometry behaviors of Yb^3+^ and Ho^3+^ co-doped GYTO crystal

**DOI:** 10.1007/s12200-023-00083-2

**Published:** 2023-10-31

**Authors:** Chuancheng Zhang, Shoujun Ding, Miaomiao Wang, Hao Ren, Xubing Tang, Yong Zou, Renqin Dou, Wenpeng Liu

**Affiliations:** 1 School of Microelectronics and Data Science, Anhui University of Technology, Maanshan, 243002 China; 2 Anhui Institute of Optics and Fine Mechanics, Chinese Academy of Sciences, Hefei, 230031 China; 3 Advanced Laser Technology Laboratory of Anhui Province, Hefei, 230037 China; 4 Anhui Provincial Joint Key Laboratory of Disciplines for Industrial Big Data Analysis and Intelligent Decision, Maanshan, 243002 China

**Keywords:** Yb,Ho:GYTO, Optical temperature sensor, Luminescence intensity ratio, Upconversion luminescence

## Abstract

Optical thermometry based on the upconversion (UC) luminescence intensity ratio (LIR) has attracted considerable attention because of its feasibility for achievement of accurate non-contact temperature measurement. Compared with traditional UC phosphors, optical thermometry based on UC single crystals can achieve faster response and higher sensitivity due to the stability and high thermal conductivity of the single crystals. In this study, a high-quality 5 at% Yb^3+^ and 1 at% Ho^3+^ co-doped Gd_0.74_Y_0.2_TaO_4_ single crystal was grown by the Czochralski (Cz) method, and the structure of the as-grown crystal was characterized. Importantly, the UC luminescent properties and optical thermometry behaviors of this crystal were revealed. Under 980 nm wavelength excitation, green and red UC luminescence lines at 550 and 650 nm and corresponding to the ^5^F_4_/^5^S_2_ → ^5^I_8_ and ^5^F_5_ → ^5^I_8_ transitions of Ho^3+^, respectively, were observed. The green and red UC emissions involved a two-photon mechanism, as evidenced by the analysis of power-dependent UC emission spectra. The temperature-dependent UC emission spectra were measured in the temperature range of 330–660 K to assess the optical temperature sensing behavior. At 660 K, the maximum relative sensing sensitivity (*S*_r_) was determined to be 0.0037 K^−1^. These results highlight the significant potential of Yb,Ho:GYTO single crystal for optical temperature sensors.

**Graphical abstract:**

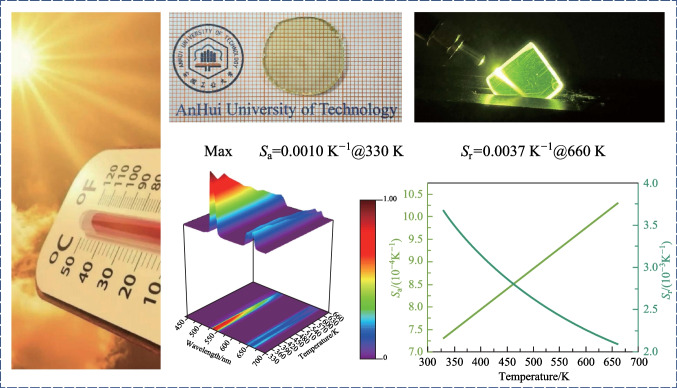

## Introduction

Lanthanide-based upconversion (UC) luminescence is a process that converts low-energy photons (near infra-red, NIR) into high-energy photons (visible) through the anti-Stokes mechanism. This phenomenon offers potential for remarkable applications in various fields, including super-resolution nanomicroscopes, photovoltaic cells, sensing, and detection [1–3]. The advantages of UC luminescence include a low autofluorescence background, excellent photostability and non-contact thermometry [4–6]. Temperature, as a fundamental and significant physical parameter, plays a crucial role in numerous aspects of our lives. Temperature control is essential in experimental settings and manufacturing processes. However, conventional temperature measurement methods, which involve contact, are not suitable for certain fields such as intracellular temperature measurement, and in coalmines or power stations due to harsh conditions [7–9]. Therefore, the development of non-contact thermometry measurements has become an important requirement.

In recent years, non-contact temperature sensing techniques based on rare earth (Re) materials have emerged as a hot topic due to their rapid response and high precision [10, 11]. Among these techniques, the luminescence intensity ratio (LIR) derived from UC luminescence is widely used for non-contact temperature measurements. Traditionally, the LIR technique relies on detecting the thermally coupled two energy levels of rare earth ions [7], such as the well-known Er^3+^(^2^H_11/2_, ^3^S_3/2_) [12] and Tm^3+^(^3^F_2,3_, ^3^H_4_) [13]. However, the sensitivity of these sensors is limited by the energy gaps between these pairs of levels [14]. As a result, non-thermally coupled energy levels (NTCLs), such as Ho^3+^(^5^F_4_/^5^S_2_ and ^5^F_5_ [15]), have gradually gained more attention of researchers. The energy levels of trivalent holmium ions (Ho^3+^) offer favorable conditions for achieving accurate temperature measurements. The energy gap equating to approximately 3000 cm^−1^ between ^5^F_4_/^5^S_2_ and ^5^F_5_ allows for distinct emission bands [16, 17]. However, commercially available 980 nm laser diodes (LDs) are not efficient at exciting Ho^3+^ ions [18]. That is why Yb^3+^ co-doping with Ho^3+^ becomes crucial, as Yb^3+^ ions play a vital role in enhancing the emission by Ho^3+^. Yb^3+^ possesses unique features, including outstanding absorption at 980 nm and favorable energy levels matching with those of Ho^3+^ ions, making it an excellent sensitizer [19–22]. The energy transfer mechanism between Yb^3+^ and Ho^3+^ in *α*-NaYF_4_ with strong green emission and in core–shell *β*-NaYF_4_ nanoparticles with red emission was investigated by Zhang et al. [23] and Pilch et al. [24]. Additionally, numerous UC luminescent matrices co-doped with Yb^3+^ and Ho^3+^ were thoroughly studied for their potential application in non-thermally coupled temperature sensors based on the LIR technique. The examples of such matrices include Y_2_Ti_2_O_7_ [25], (La_0.1_Y_0.9_)_2_O_3_ [26], Y_2_O_3_ [27], GaF_2_ [28], KLu(WO_4_)_2_ [29] and fluoroborate glasses [30]. The RE tantalate series matrices have witnessed rapid development since their first synthesis by Brixner in 1964 [31]. These matrices offer excellent physical and chemical stability which is extremely valuable [32].

The low symmetry and strong crystal field effect of GdTaO_4_ (GTO) crystal may lead to a higher probability of electro-dipole transitions which is an attractive property in enhancing the photoluminescence efficiency of Re ions [33]. As a result, the 2.1 μm GTO laser crystal doped with Ho^3+^ and Yb^3+^/Ho^3+^ co-doped Gd_*x*_Y_1−*x*_TaO_4_ (GYTO) crystal with the output wavelength of 2.9 μm have been realized by Zhang et al. [33] and Dou et al. [34]. However, there are scarce reports regarding the UC luminescence and temperature sensor application of Yb^3+^/Ho^3+^ co-doped GTO or GYTO crystals. The ionic radius of Y^3+^ (0.893 Å) is slightly smaller than that of Gd^3+^ (0.938 Å), and so the introduction of Y^3+^ does not cause significant lattice mismatch or structural changes of GTO. Instead, the impurity Y^3+^ efficiently distorts the local symmetry of Gd^3+^ and tunes the crystal field of GTO [34], further amplifying the merits of this crystal. Therefore, the objective of this paper is to investigate the UC luminescence of Yb^3+^/Ho^3+^ co-doped GYTO crystal and explore its potential applications in temperature sensing.

## Experimental section

### Preparation of samples

**Fig. 1:**
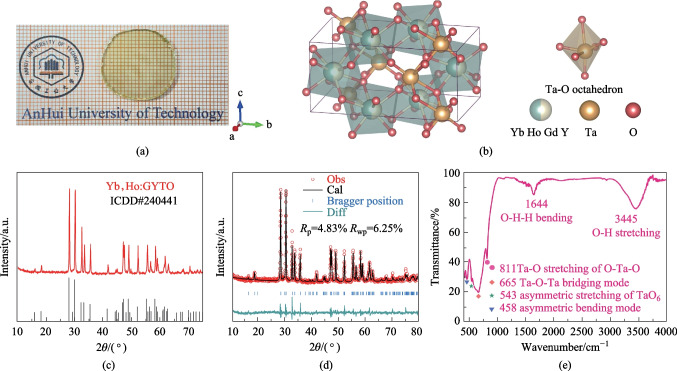
Structure characterization of Yb,Ho:GYTO crystal: **a** single crystal plate of Yb,Ho:GYTO after cutting and polishing, **b** crystal structure of Yb,Ho:GYTO, **c** XRD pattern of Yb,Ho:GYTO, **d** Rietveld refinement XRD patterns of Yb,Ho:GYTO and **e** the FT-IR spectra of Yb,Ho:GYTO crystal

A high-quality GYTO crystal, co-doped with 5 at% Yb^3+^ and 1 at% Ho^3+^, was grown in N_2_ atmosphere using the Czochralski (Cz) method with the JGD-400 Cz furnace (CETC). The crystal growth utilized raw materials Yb_2_O_3_, Ta_2_O_5_, Gd_2_O_3_, Ho_2_O_3_, and Y_2_O_3,_ all with a purity of 4N and purchased from Shanghai Aladdin Biochemical Technology Co., Ltd. The growth mechanisms employed in this study were consistent with our previous research [35]. The plate presented in Fig. 1a, with a thickness of 2 mm, was cut from the as-grown crystal and polished. The absence of cracks and inclusions in the plate indicated the exceptional optical quality of the crystal, which is crucial for its application in transmittance and absorption characterization.

### Characterizations

The structural characterization of as-grown crystal was conducted using a Bruker D8 Advance X-ray diffractometer equipped with Cu-K*α* radiation (*λ* = 1.5406 Å). The diffraction angle (2*θ*) ranged from 10° to 80° with a step size of 0.02°. Fourier transform infrared (FT-IR) spectra were obtained using a Nicolet 6700 spectrometer. Morphology and element distribution were analyzed using a JSM-6510 scanning electron microscope (SEM). The UC emission spectra were measured using an Omni-λ5028i spectrometer coupled with an Andor DU401-BVF charge coupler. A semiconductor laser with a maximum output power of 10 W at 980 nm (BWT Beijing Ltd.) was employed as an excitation source. Temperature-dependent spectra were recorded using the same spectrometer equipped with a temperature regulator that provided a control precision of 0.5 K.

## Results and discussion

### Crystal structure

To investigate the phase purity and crystalline nature of the crystal, X-ray diffraction (XRD) and Rietveld refinement were conducted. Figure 1c displays a comparison between the XRD patterns of the crystal and standard card GTO (ICDD#240441 [36]). The diffraction peaks of the sample align closely with those of GTO, indicating the successful attainment of a pure phase GYTO crystal with negligible impurities. For a more in-depth analysis of the unit cell parameters and for coordination of the crystal, Rietveld refinement was performed using the Fullprof software. An initial model of M-type GTO was employed. The refined patterns and main crystallographic parameters can be observed in Fig. 1d and Table 1, respectively. It is worth emphasizing that the ionic radii of Y^3+^ (0.90 Å), Yb^3+^ (0.85 Å), and Ho^3+^ (0.89 Å) are all smaller than that of Gd^3+^ (0.93 Å). Consequently, the actual unit cell parameters of the as-grown crystal were expected to be smaller than those of GTO crystal (*a* = 5.405 ± 0.002 Å, *b* = 11.063 ± 0.007 Å, *c* = 5.084 ± 0.005 Å) [37] in theory. The refined results unambiguously confirm this conclusion. The reliability coefficients, *R*_p_ = 4.83% and *R*_wp_ = 6.25%, indicate that the crystal exhibits a monoclinic phase (M-type), with Ta^3+^ coordinated by six O^2−^ ions, forming a distorted octahedron [38]. The crystal structure, drawn in Fig. 1b, as created using VESTA software, reveals that the Gd^3+^, Y^3+^, Yb^3+^, and Ho^3+^ ions are randomly distributed on the 4e Wyckoff site, and this leads to the distortion of local crystal filed of Gd^3+^ and effectively enhances the luminescence of Ho^3+^. The FT-IR spectrum provides a reliable method for determining phonon energy and unveiling various structural details of samples. In Fig. 1e, the FT-IR spectra of Yb,Ho:GTYO crystal is presented. The four characteristic peaks observed at 811, 665, 543, and 458 cm^−1^ are consistent with those reported of GdTaO_4_ in Ref. [39]. This further corroborates the fact that the as-grown crystal is of a pure monoclinic phase structure.

**Table 1:** Rietveld refined structural parameters of the Yb,Ho:GYTO crystal

Formula	Yb^3+^,Ho^3+^:Gd_0.74_Y_0.2_TaO_4_
Crystal system	Monoclinic
Space group	*I* 2/*a*
Cell parameters	*a* = 5.385(1) Å, *b* = 11.031(3) Å, *c* = 5.080(4) Å *α* = *γ* = 90°, *β* = 95.5792°
Reliability	*R*_p_ = 4.83%, *R*_wp_ = 6.25%,* χ*^*2*^ = 3.02

**Fig. 2:**
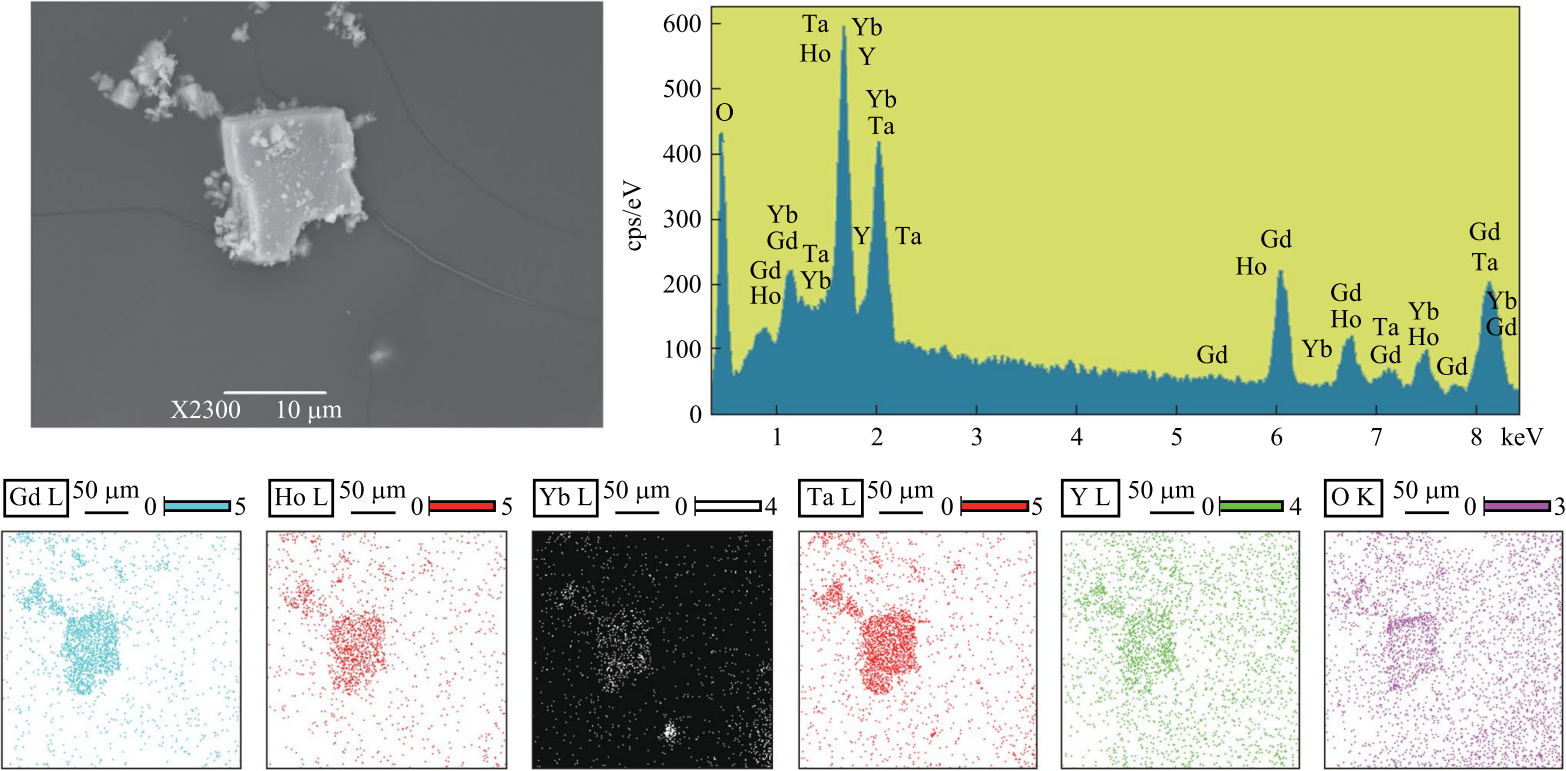
SEM, EDS images and elemental mapping images of Yb,Ho:GYTO

To ascertain the composition of Yb,Ho:GYTO, SEM was employed, and the resulting element mapping photos are given in Fig. 2. The presence of characteristic peaks corresponding to Gd, Y, Ta, and O in the EDS images confirms the successful growth of GYTO crystal. Analysis of the mapping images unambiguously identifies the presence of Yb, Ho, Gd, Ta, Y, and O elements uniformly distributed throughout the sample.

### Upconversion luminescence

**Fig. 3:**
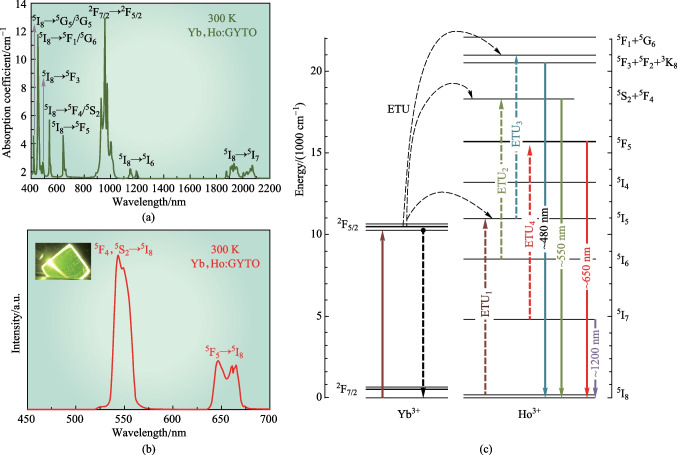
Room temperature spectroscopic properties of Yb,Ho:GYTO crystal: **a** room temperature absorption spectrum, **b** room temperature emission spectrum and **c** the energy levels of Yb and Ho and the possible UC processes

The absorption spectra of Yb,Ho:GYTO in the range of 400–2200 nm at room temperature are shown in Fig. 3a. Among the eight peaks observed, the most prominent and broadest peak is located at 957 nm, which corresponds to the characteristic absorption of Yb^3+^ and corresponds to the ^2^F_7/2_ → ^2^F_5/2_ transition. This absorption band is well aligned with the commercially available InGaAs LD. Additionally, the broad absorption band facilitates improved pumping efficiency and reduced temperature dependence of the pumping source. The remaining peaks, found at 1931, 1150, 642, 539, 487, 450, and 419 nm, can be attributed to the transitions of Ho^3+^ (^5^I_8_ → ^5^I_7_, ^5^I_8_ → ^5^I_6_, ^5^I_8_ → ^5^F_5_, ^5^I_8_ → ^5^F_4_/^5^S_2_, ^5^I_8_ → ^5^F_3_, ^5^I_8_ → ^5^F_1_/^5^G_6_, ^5^I_8_ → ^5^G_5_/^3^G_5_) [40–42]. The efficiency of UC is significantly influenced by the rate of multiphonon nonradiative relaxation (MNR), represented by *W*_NR_, and can be expressed as [43]

1 $$\begin{array}{c}{{W}_{\mathrm{NR}}\propto \left[1-\mathrm{exp}\left(-\frac{\hbar w}{{k}_{\mathrm{B}}T}\right)\right]}^{-P},\end{array}$$

where *k*_B_*,* $\hbar$*w, T*, and *P* are the Boltzmann constant, the phonon energy of the matrix, the absolute temperature, and the number of phonons to complement MNR, respectively. The *P* value can be calculated by

2 $$\begin{array}{c}P=\frac{\Delta E}{\hbar w}.\end{array}$$

These two formulas indicate that smaller MNR always follows the matrix with lower phonon energy. The quite low phonon energy of GTO (~ 345 cm^−1^ [44]) which is comparable with that of tetrafluoride (~ 350 cm^−1^ [45]) is conducive to achieving lower MNR. In this way, the Ho^3+^ ions in GYTO matrix may be of great potential for reaching higher UC luminescence efficiency.

The room temperature UC emission spectra of Yb,Ho:GYTO crystal under 980 nm excitation are presented in Fig. 3b. The inserted image clearly shows a remarkable green emission that is visible to the naked eye. The green emission at approximately 550 nm and the red emission at 650 nm correspond to characteristic transitions of Ho^3+^: ^5^F_4_/^5^S_2_ → ^5^I_8_ and ^5^F_5_ → ^5^I_8_, respectively [20, 46, 47]. Previous studies [23, 26] have extensively investigated the phenomenon of concentration quenching in UC emission, primarily attributed to an excessive concentration of Yb^3+^. In these studies, it was shown that, when the concentration of Yb^3+^ exceeds 10 at%, the concentration quenching becomes evident, leading to a significant decrease in UC emission. To avoid such quenching effects and ensure the highest energy transfer efficiency between Yb^3+^ and Ho^3+^, we controlled the concentration of Yb^3+^ at approximately 5 at%. In Fig. 3c, the energy levels of Yb^3+^ and Ho^3+^ are illustrated to elucidate the UC emission mechanism. Employment of ground-state absorption (GSA) with InGaAs LD, Yb^3+^ achieves population inversion. Subsequently, the UC emission of Ho^3+^ is realized through consecutive energy transfer with the assistance of photons (ETU). In the case of the strong green emission resulting from the ^5^S_2_/^5^F_4_ → ^5^I_8_ transition of Ho^3+^, the ground state ^5^I_8_ is excited to ^5^F_3_/^5^F_2_/^3^K_8_ through ETU_1_ and ETU_3_, ^2^F_5/2_(Yb^3+^) + ^2^F_5/2_(Yb^3+^) + ^5^I_8_(Ho^3+^) → ^5^F_3_/^5^F_2_/3K_8_(Ho^3+^) + ^2^F_7/2_(Yb^3+^) + ^2^F_7/2_(Yb^3+^). These ions then relax to the ^5^S_2_/^5^F_4_ level via nonradiative relaxation (NR). Additionally, the ions in the ^5^S_2_/^5^F_4_ level may also originate from those at the ^5^I_6_ level, which relaxes from ^5^I_5_ then accepting energy from another Yb^3+^ through ETU_2_^2^F_5/2_(Yb^3+^) + ^5^I_6_(Ho^3+^) → ^5^S_2_/^5^F_4_(Ho^3+^) + ^2^F_7/2_(Yb^3+^). The red emission arises from further NR of ^5^I_6_ to the ^5^I_7_ level, followed by excitation to ^5^F_5_ through ETU_4_^2^F_5/2_(Yb^3+^) + ^5^I_7_(Ho^3+^) → ^5^F_5_(Ho^3+^) + ^2^F_7/2_(Yb^3+^). Finally, the transition from ^5^F_5_ to ^5^I_8_ generates the red emission centered at 650 nm. In addition, the NR process from the ^5^S_2_/^5^F_4_ level to ^5^F_5_ of Ho^3+^ ions can also induce this red emission. It should be noted that the energy gaps between ^5^S_2_/^5^F_4_ and ^5^F_5_, ^5^I_6_ and ^5^I_7_, are approximately 2931 and 3500 cm^−1^, respectively [48]. Thus, realizing MNR would require at least 8–10 intrinsic GYTO phonons, which is not realistic. Nonetheless, resonant cross-relaxation (CR) among adjacent Ho^3+^ ions may participate in populating the intermediate energy levels and enhance the populations of ^5^S_2_/^5^F_4_ and ^5^F_5_ [49].

**Fig. 4:**
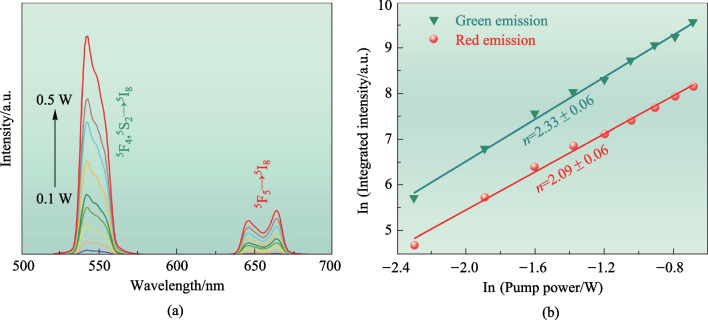
Power-dependent UC luminescence properties of Yb,Ho:GYTO crystal: **a** UC emission spectra of Yb,Ho:GYTO crystal at different excitation powers and **b** ln–ln plots of pump power dependence of the UC emission intensity

The emission spectra of this crystal were investigated under 980 nm excitation with varying power in order to further explore the mechanism behind UC emission. Figure 4a clearly shows that the intensity of green and red emissions increases consistently as the pump power increases from 0.1 to 0.5 W. This observation suggests that the population in the ^5^F_5_ level of Yb^3+^ effectively enhances the number of excited states of Ho^3+^ through energy transfer upconversion (ETU). Notably, the shape of the emission bands and peak positions remain unchanged. It is well-known that the integrated emission intensity and the pump power serve as valuable indicators for studying the UC luminescence mechanism of Ho^3+^. Consequently, investigating the absorption processes during the UC mechanism is of utmost importance for gaining a deeper understanding of the luminescence mechanism.

The relationship between integrated intensity *I*_up_, pump power *P*, and the number of photons can be expressed as [50]

3 $$\begin{array}{c}{I}_{\mathrm{up}}\propto {P}^{n}.\end{array}$$

From this equation, another logarithmic relationship can be deduced

4 $$\begin{array}{c}{\rm{ln}}{I}_{\mathrm{up}}=n{\rm{ln}}P+C.\end{array}$$

The use of a logarithmic–logarithmic (ln–ln) plot is advantageous for depicting the dependence of UC emission intensity (*I*_up_) on the *P* variable. Figure 4b illustrates that the experimental data can be accurately fitted by a linear function. The values of *n* for the Yb,Ho:GYTO crystal, corresponding to green and red emissions, are determined to be 2.33 and 2.09, respectively. These values indicate that both the green and red UC emissions adhere to a two-photon mechanism.

### Temperature sensing behavior

**Fig. 5:**
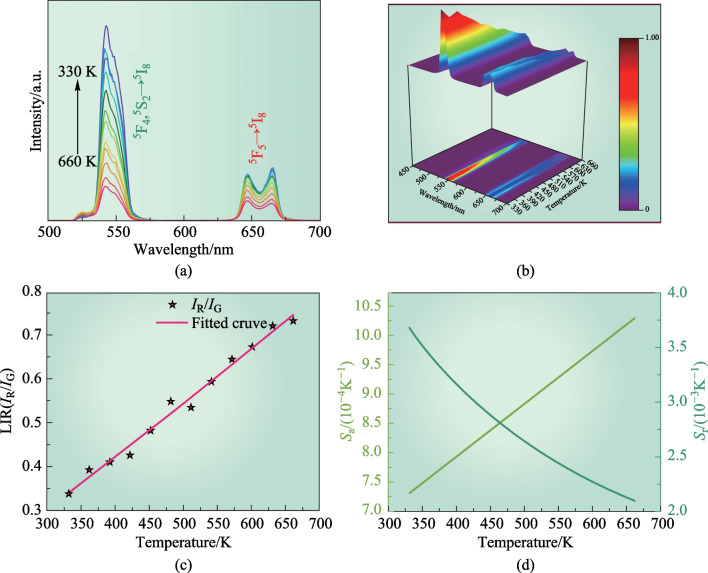
Temperature sensing behavior of Yb,Ho:GYTO crystal: **a** temperature-dependent UC emission spectra, **b** emission spectrum of Yb,Ho:GYTO at different temperatures (330–660 K), **c** LIR values as a function of temperature ranging from 330 to 660 K and **d** temperature-dependent absolute and relative sensitivity ranging from 330 to 660 K

To thoroughly investigate the temperature-sensing behavior of Yb,Ho:GYTO crystal, the temperature-dependent non-thermally coupled energy levels (NTCLs) UC emission spectra were meticulously examined. Figures 5a, b display the UC luminescence spectra over the 330–660 K range, clearly demonstrating a consistent decrease of both the green and red emission intensities with increasing temperature. Notably, the decline in green UC emission intensity is more pronounced, as seen in Fig. 5c. This phenomenon is further supported by the observation that the luminescence intensity ratio (LIR) of red and green (*I*_R_/*I*_G_) progressively increases from 330 to 660 K. This effect may arise from the heightened probability of MNR from the ^5^S_2_/^5^F_4_ to ^5^F_5_ energy level due to the elevated phonon energy of the crystal with increasing temperature. To elucidate the relationship between red and green emissions, the LIR of both colors is employed. It is important to note that the green and red UC emissions of Ho^3+^ originate from non-thermally coupled energy levels (^5^S_2_/^5^F_4_ and ^5^F_5_); electrons cannot populate the levels through thermal excitation, because of the large energy gaps involved. Therefore, the temperature-dependent LIR can be expressed as [26]

5 $$\begin{array}{c}{\rm{LIR}}\left(\frac{{I}_{\mathrm{R}}}{{I}_{\mathrm{G}}}\right)=A+B\times T+C\times {T}^{2}.\end{array}$$

The formula presented includes constants denoted by *A*, *B*, and *C*, while *T* represents the absolute temperature. Figure 5c shows the temperature dependence of the LIR for the Ho,Yb:GYTO crystal in the range of 330–660 K. The fitted curve aligns well with the experimental data, revealing an increase in LIR values across the temperature range of 330–660 K. This observation suggests that this crystal has a potential optical thermometry application in wide temperature range. To assess the sensor’s performance, further investigation into absolute sensitivity (*S*_a_) and relative sensitivity (*S*_r_) was conducted. The relationship between *S*_a_, *S*_r_, and temperature can be expressed as follows

6 $$\begin{array}{c}{S}_{\mathrm{a}}=\left|\frac{d\left({\rm{LIR}}\right)}{d\left(T\right)}\right|,\end{array}$$

7 $$\begin{array}{c}{S}_{\mathrm{r}}=\left|\frac{d\left({\rm{LIR}}\right)}{d\left(T\right)}\frac{1}{\rm{LIR}}\right|.\end{array}$$

**Fig. 6:**
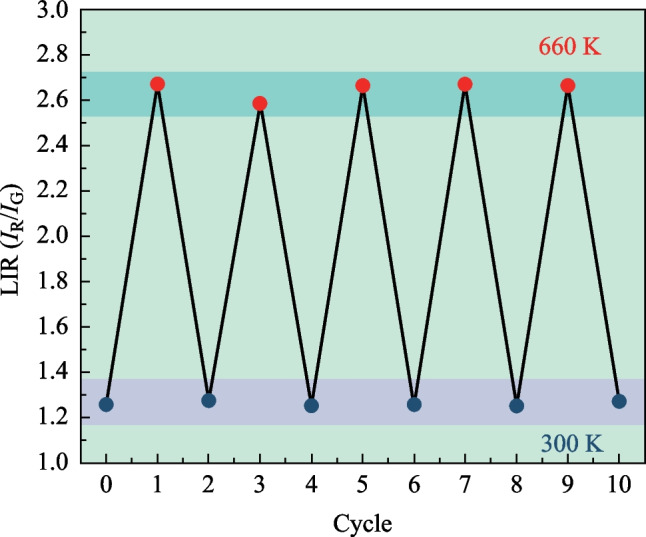
Thermometer reliability of the crystal during multiple heating–cooling cycles in the temperature range of 300–660 K

The* S*_a_ and* S*_r_ values were calculated using Eqs. (6) and (7), respectively, and the corresponding results are presented in Fig. 5d. It is evident from the figure that *S*_a_ exhibits a monotonic increase as the temperature ranges from 330 to 660 K. However, the *S*_r_ variation follows a contrasting trend. The maximum values observed for *S*_a_ and *S*_r_ are 1.03 × 10^−3^ K at 660 K and 3.67 × 10^−3^ K at 330 K, respectively. Table 2 provides an overview of the optical thermometry performance of various Yb^3+^/Ho^3+^ co-doped materials. Notably, this crystal demonstrates excellent temperature sensing capabilities, as indicated by its high *S*_r_ value of 3.67 × 10^−3^ K, surpassing several other Ho^3+^ doped materials, such as Yb^3+^,Ho^3+^:glass ceramics (1.00 × 10^−3^ K), Yb^3+^,Ho^3+^:GaF_2_ crystalline powders (3.00 × 10^−3^ K), and Yb^3+^,Ho^3+^:Y_2_Ti_2_O_7_ nanotubes (2.50 × 10^−3^ K). Furthermore, this crystal exhibits good thermometric reliability during multiple heating–cooling cycles, as shown in Fig. 6. In total, these findings highlight the crystal’s significant potential for application in optical thermometry.

**Table 2:** *S*_r_ and *S*_a_ values of various Yb^3+^/Ho^3+^ co-doped materials

Materials	*S*_a_/K^−1^	*S*_r_/K^−1^	*λ*_ex_/nm	Δ*T*/K	Refs.
Yb^3+^,Ho^3+^:GYTO	0.0010	0.0037	980	330−660	This work
Yb^3+^,Ho^3+^:Y_2_O_3_	–	0.0038	980	293−873	[51]
Yb^3+^,Ho^3+^:GaF_2_	–	0.0030	980	473−713	[52]
Yb^3+^,Ho^3+^:KLu(WO_4_)_2_	0.0038	0.0054	980	297−673	[53]
Yb^3+^,Ho^3+^:Y_2_Ti_2_O_7_ Nd^3+^,Yb^3+^,Ho^3+^:LaNbO_4_ Yb^3+^,Ho^3+^:Sc_2_Mo_3_O_12_	0.0006 – 0.0002	0.0025 0.0020 0.0085	980 808 980	303−633 303–693 303−573	[25] [54] [55]

## Conclusion

In summary, the high optical quality Yb,Ho:GYTO crystal was grown using the Cz method. The crystal structure was characterized through X-ray diffraction, Fourier transform infrared spectroscopy and scanning electron microscopy. The results revealed that Yb,Ho:GYTO crystallizes in a Monoclinic (M type) structure with a space group of *I* 2/*a*. The incorporation of Yb^3+^ and Ho^3+^ ions did not alter the GYTO matrix structure type. Notably, when irradiated with a 980 nm LD, a vivid green UC emission was observed with the naked eye. The green and red UC emissions from two non-thermally coupled energy levels in Ho^3+^ were attributed to a two-photon mechanism, as evidenced by the analysis of power-dependent UC emission spectra. The optical temperature sensing capabilities of Yb,Ho:GYTO were investigated using LIR technology and temperature-dependent UC emission spectra. The maximum absolute sensing sensitivity (*S*_a_) and relative sensing sensitivity (*S*_r_) were calculated as 0.0010 and 0.0037 K^−1^, respectively. These findings highlight the significant potential of Yb,Ho:GYTO for applications in optical thermometry.

## Data Availability

The data that support the findings of this study are available from the corresponding authors, upon reasonable request.
